# The impact of hearing impairment on early academic achievement in Aboriginal children living in remote Australia: a data linkage study

**DOI:** 10.1186/s12889-020-09620-6

**Published:** 2020-10-07

**Authors:** Jiunn-Yih Su, Steven Guthridge, Vincent Yaofeng He, Damien Howard, Amanda Jane Leach

**Affiliations:** 1 Centre for Child Development and Education, Menzies School of Health Research, Charles Darwin University, Darwin, Australia; 2 Child Health Division, Menzies School of Health Research, Charles Darwin University, Darwin, Australia; 3 Phoenix Consulting, Darwin, Australia

**Keywords:** Otitis media, Conductive hearing loss, Academic achievement, Data linkage, Aboriginal children, NAPLAN

## Abstract

**Background:**

The prevalence of otitis media (OM) and related hearing loss has remained persistently high among some groups of Australian Aboriginal children who are also reported to have poor academic outcomes. The general literature remains inconclusive about the association between OM-related hearing loss and academic performance in primary school. This study aimed to investigate this association in Aboriginal children living in the Northern Territory (NT) of Australia.

**Methods:**

A retrospective, observational cohort study was conducted for 2208 NT Aboriginal children, aged about 8 years, living in remote and very remote communities. The explanatory variable was audiometrically determined hearing level as recorded in the Remote Hearing Assessment dataset. The outcome variable consisted of scale scores in the five domains of the National Assessment Program – Literacy and Numeracy (NAPLAN) for Year 3. Other linked datasets used in the study included school attendance records, perinatal records and community level information on relative remoteness, socioeconomic disadvantage and housing crowdedness. Fixed effects linear regression models were used for statistical analyses.

**Results:**

Compared with children with normal hearing and after controlling for a range of covariates, children with mild hearing impairment (HI) scored lower in Writing and Spelling by 15.0 points (95% CI: − 22.4 to − 7.6, *p* < 0.0005) and 5.0 points (95% CI: − 9.6 to − 0.3, *p* = 0.037), equivalent to 7.3 and 2.1% of the mean score, respectively. Children with moderate or worse HI scored lower in Writing and Numeracy by 13.4 points (95% CI, − 24.8 to − 1.9, *p* = 0.022) and 15.2 points (95% CI, − 27.6 to − 2.7, *p* = 0.017), both equivalent to 6.3% of the mean score the respective domain. Other factors associated with poorer NAPLAN results included being male, lower Year 2 school attendance, low birthweight, average household size> 5 persons, living in a very remote community and speaking English as a second language.

**Conclusions:**

OM-related HI was independently associated with poorer early year academic achievement in Aboriginal children living in remote NT communities. Interventions to improve academic outcomes for Aboriginal children must incorporate actions to address the negative impact associated with HI through early detection, effective treatment and ongoing support for affected children.

## Background

Conductive hearing loss is the most common complication of middle ear infection, also called otitis media (OM) [1, 2]. Although some studies showed hearing loss in young children can adversely affect language development and literacy skills [3, 4], as well as academic performance [5–8], overall the literature remains inconclusive about the extent to which conductive hearing loss associated with OM influences academic performance in primary school [9–11]. The impact of hearing loss on educational performance can be mediated in two ways: firstly, OM and hearing loss may persist from early childhood to the school years; or secondly, hearing loss during critical periods of neurological development in early childhood may result in difficulties with auditory processing skills [12]. In this latter case a child’s hearing may return to normal but auditory processing problems still compromise learning, especially in noisy classrooms.

In the case of Australian Aboriginal children, several factors have contributed to the paucity of available evidence. The prevalence of OM has remained persistently high among Aboriginal children (as high as 90% in some studies) [13, 14], which has presented difficulties in finding control groups with normal hearing. In contrast to non-Aboriginal children, OM in Aboriginal children commonly develops early in life (within 3 months of birth) and is prone to recur repeatedly through childhood, and even into adolescence [15, 16]. The clinical presentation and severity of OM in this population is also more varied, including a much greater proportion of cases progressing to chronic suppurative OM [15]. The situation is further complicated by the fluctuating nature of the OM-related hearing loss which can vary over time between mild, moderate and severe hearing loss, and can be either transient or persisting [10, 17]. Misclassification can easily occur when studies fail to assess the duration and severity of OM, or the degree of hearing loss [9]. The lack of population-level, hearing assessment data has also limited investigations. In the Northern Territory (NT) of Australia, the lack of hearing assessment data is, in part, the result of a majority of NT Aboriginal children living in remote communities where there is a high turnover of health care staff [18] and limited access and long waiting times for specialist ear health services [19]. Even when specialist ear health services are provided locally, they have not been accessed universally [20].

Methodological differences between studies, including varied methods of diagnosis and varied definitions of hearing, have also led to differences in the reported findings [21, 22]. Analytic methods for population-level studies also need to take account of correlation within families or communities and a range of other contextual factors [9]. Contextual factors previously reported to adversely affect Aboriginal children’s academic performance include socioeconomic disadvantage, the enduring impact of colonisation, overcrowded housing, general ill-health and parental factors (such as literacy and education attainment and family violence) [23–25].

This population level study aimed to investigate the association between OM-related conductive hearing loss and early academic achievement for Aboriginal children living in remote communities in the NT. It was made feasible through the recent unit-level linkage of information from the Remote Hearing Assessment dataset and a range of health and education administrative datasets.

## Methods

### Study design and participants

Undertaken as part of the Hearing Loss in Kids (HeloKids) Project [26], this was a retrospective observational study with a study cohort of NT-born Aboriginal children with linked records in four key administrative datasets. The four datasets were contained within a comprehensive, de-identified data repository containing a total of fourteen administrative datasets for NT children [27]. The linkage was undertaken by SA NT DataLink using a combination of probabilistic linkage and clerical review of uncertain matches [28]. The four key datasets were:

- NT Perinatal Data Register: was established in 1986 and is a statutory collection of maternal and perinatal information for all births in the NT.
- School dataset: an administrative dataset containing enrolment and daily attendance records for students attending NT Government schools over the period 2005–2016.
- National Assessment Program – Literacy and Numeracy (NAPLAN) dataset: is a national collection, with the research datasets containing individual test results and related statistics for all participating children attending school in the NT at the time of assessments in Years 3, 5, 7 and 9.
- Remote Hearing Assessment (RHA) database: contains individual clinical and audiometric assessment records collected, from 2007 onwards, by the NT Outreach Hearing Health Program. The program provides specialist hearing health services to Aboriginal children living in remote and very remote NT communities, which are all towns and communities outside the urban centre of Darwin and surrounding area [20]. Other hearing assessment services were available for children living in Darwin and data were not available for this study.

Children who underwent surgical treatment for OM before the age of 4 years were excluded from the study cohort because surgery might have altered the impact of hearing loss during early childhood. This step was undertaken by linking data for children in the study cohort to a fifth dataset, the NT Hospital Separations dataset, and excluding children with a record of admission before age 4 with a diagnosis code for OM and a related surgical procedure code (coded using the International Statistical Classification of Diseases and Related Health Problems, Tenth Revision, Australian Modified and listed in Additional file 1 Table 1.)

### The explanatory variable

The explanatory variable was level of hearing, based on the results of audiologist-performed audiometric hearing assessment recorded in the RHA database. The results were the average threshold of hearing (as deviation from the normal threshold, in decibels hearing level [dB HL]) for the three frequencies: 500 hertz (Hz), 1000 Hz and 2000 Hz, as assessed with pure tone audiometry. The RHA result for each ear was classified as either normal or one of four levels of hearing loss, namely mild (16–30 dB HL), moderate (31–60 dB HL), severe (61–90 dB HL) and profound (≥ 91 dB HL), a comparatively conservative classification which has been deemed more suitable for children aged under 15 years [20]. Only results of conductive and mixed hearing loss were included in the study.

Based on the RHA results, in two ears, the definition of level of hearing was classified as one of four categories:

- ▪ Normal hearing: normal audiometry results in both ears.
- ▪ Unilateral hearing loss: normal in one ear and any degree of hearing loss in the other ear.
- ▪ Mild hearing impairment (HI): mild hearing loss (16–30 dB HL) in the better hearing ear.
- Moderate or worse HI: moderate or worse hearing loss (> 30 dB HL) in the better hearing ear.

Among children in the study cohort, 75% of children had their first recorded hearing assessment performed when aged 5 years or older and 51% had multiple assessments. OM in NT Aboriginal children tends to develop very early in life, be persistent and often asymptomatic [16, 29], however it is often not diagnosed until an older age. We therefore assumed that the first recorded audiometry result was representative of a child’s past hearing level regardless of the age at time of assessment.

### Outcome variables

Academic achievement was measured using student’s NAPLAN test results. NAPLAN tests are held annually, in all Australian schools, for students in Year 3, 5, 7 and 9. The tests assess student’s performance in five domains: reading, writing, spelling, grammar and punctuation, and numeracy. The NAPLAN is designed to assess a student’s understanding of the core elements of the national curriculum and is an aid in identifying those students who may not have attained the skills required to progress to the next year of schooling [30]. For each test domain, the raw scores were converted to NAPLAN scale scores, which ranged from 0 to 1000, so that scores can be placed on the national scale to enable comparisons of students’ performance across schools and time periods [30].

The NAPLAN dataset used in this study contained test results for all NT Government schools. This study used Year 3 results as the measure for early year academic achievement, because there was a substantially greater proportion of missing NAPLAN test results for Year 5 onwards, with many students recorded as ‘absent’. We used the scale scores for the five domains as the outcome variables, instead of the more common dichotomous result of whether or not a student’s score was below the national minimum standard (NMS). The scale scores have been used in other studies using NAPLAN results as outcome measures [31–34]. The NMS represents the benchmark for the basic level of knowledge and understanding the student requires to function at the year level [30]. Being a dichotomised variable, the NMS had the limitation of losing data robustness; further, the NMS for each domain was determined based on the performance of the national NAPLAN cohort (with < 5% Aboriginal and Torres Strait Islander students) and its use would result in a loss of discernment within the Aboriginal study cohort who have a comparatively much higher proportion of results below the NMS than the national cohort [30].

### Control variables

A range of variables that may moderate or confound the outcome of students’ academic achievements were included in the multivariate regression model building process, as listed below with their respective data source:

1. Perinatal Data Register: sex, number of antenatal visits, whether the mother was a teenager at time of birth (teenage pregnancy), maternal diabetes, maternal hypertension, smoking or alcohol consumption during pregnancy, low birthweight (less than 2500 g), preterm birth (less than 37 weeks gestational age), parity, twin birth, APGAR score at 5 min of less than 7;
2. NAPLAN dataset: age at assessment; year of NAPLAN test;
3. School data: annual attendance rates for Year 2, speaking English as a second language (ESL), ever attended preschool;
4. ABS Census data: The following community-level information was extracted from census information based on the location of the school (at level of Statistical Local Area (SLA) that a child attended in Year 2: community-level socioeconomic disadvantage (Index of Relative Socio-economic Disadvantage (IRSD) [35, 36]), level of relative remoteness (Accessibility and Remoteness Index of Australia (ARIA+, only ‘remote’ and ‘very remote’ categories in the study cohort) [36]), housing overcrowding at the community-level (average person per bedroom and average household size).

### Statistical analysis

Chi-square test and t-test were used to compare the study cohort with a group of children with data in three key datasets, but without a hearing assessment record. Univariate and multivariate linear regressions were used to estimate the association between the independent variable and outcome variables while controlling for the selected control variables. The fixed effects modelling method was used to adjust for the effects of unobserved differences between schools, such as school resources, staff turnover rates, number and quality of the teachers, school and class sizes. This method is necessary for our exclusively Aboriginal study cohort as evidenced by Australian studies showing that the Aboriginal population is not evenly distributed across schools and that characteristics of schools have an impact on Aboriginal students’ educational outcomes [31, 37]. A parsimonious model building strategy was adopted in the regression modelling process [38, 39]. The regression analyses were first undertaken at univariate level to select variables with a *p*-values < 0.25, which were fitted in the multivariate regression model building process. A number of additional variables were retained in the model based on findings of past studies [39, 40]. Unadjusted and adjusted regression coefficients (COEFs) and 95% confidence intervals (CIs) were estimated and reported for each variable in relation to a unit change in the scale score of the NAPLAN domain examined. All analyses were conducted using Stata version 15 (Stata Corporation, College Station, TX, USA).

## Results

### Descriptive statistics

**Fig. 1:**
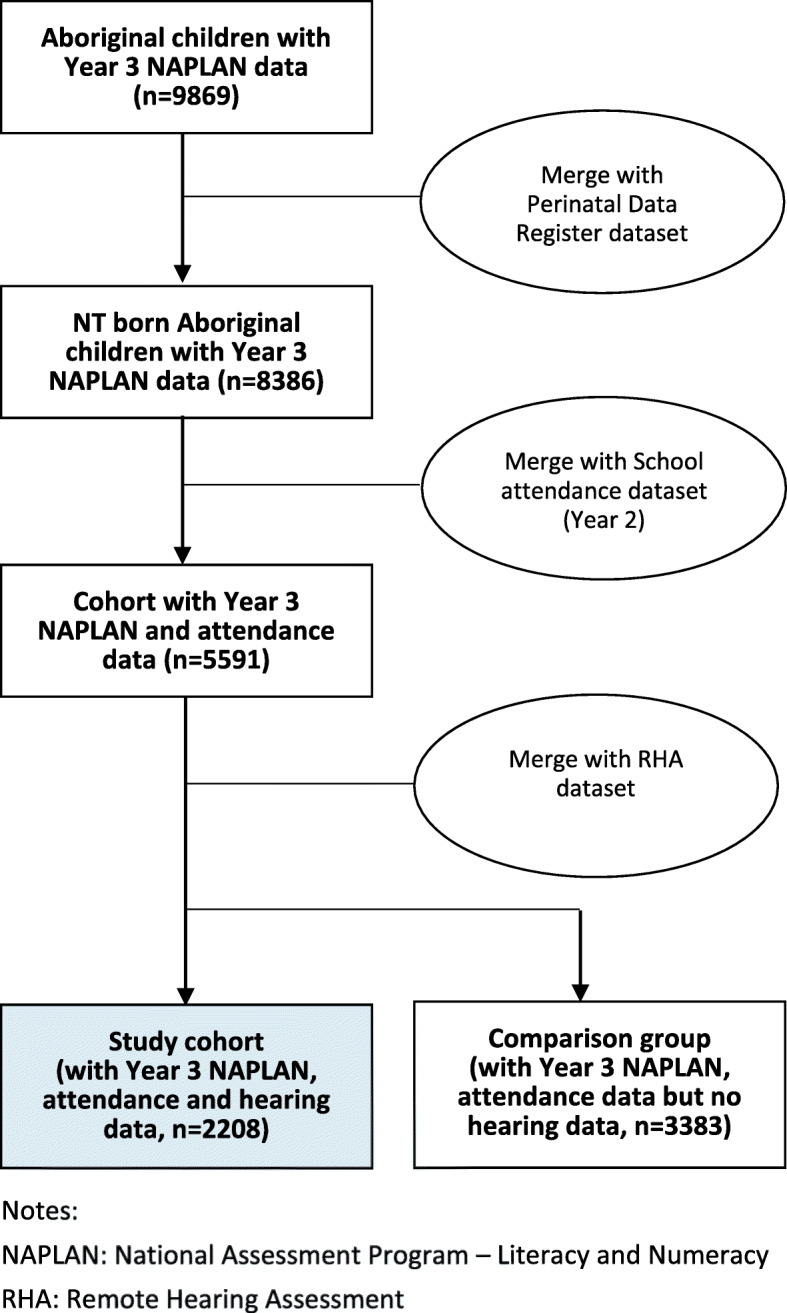
Processes of dataset merging and study cohort (shaded) selection

The study cohort was 2208 NT born Aboriginal children living in remote and very remote NT communities, who had linked Perinatal Data Register, Year 3 NAPLAN, Year 2 attendance and hearing assessment data. The selection process is summarised in Fig. 1. We compared the study cohort with the comparison group of children with data in three datasets but with no record of hearing assessment (*n* = 3383) and found evidence for difference in 6 of the 10 control variables included in the univariate analysis: the study cohort had a higher proportion of children living in very remote communities; living in conditions of crowded housing; speaking English as a second language, having records of attending preschool and having a Year 2 attendance rate of less than 80%. The mean scale score was higher in the comparison group for all five NAPLAN domains (Additional file 1 Table 2).

### Results of univariate and multivariate analyses

The univariate analysis found evidence for an association between HI and poorer academic achievement in Writing, Spelling and Numeracy (Table 1). Compared with children with normal hearing, those with mild HI scored lower in Writing and Spelling with unadjusted COEFs of − 15.8 points (95% CI: − 23.7 to − 8.0, *p* < 0.0005) and − 5.4 points (95% CI: − 10.5 to − 0.2, *p* = 0.04), respectively; and those with moderate or worse HI scored lower in Writing and Numeracy by − 12.7 points (95% CI: − 24.7 to − 0.8, *p* = 0.037) and − 16.0 points (95% CI: − 27.6 to − 2.7, *p* = 0.016).

**Table 1:** Results of univariate regression analyses for the association between the explanatory and control variables, and the scale scores of NAPLAN Year 3 tests in five domains, Northern Territory Aboriginal children, NAPLAN tests from 2009 to 2016

Variable	Reading		Writing		Grammar		Spelling		Numeracy	
	Unadj COEF	(95% CI)	Unadj COEF	(95% CI)	Unadj COEF	(95% CI)	Unadj COEF	(95% CI)	Unadj COEF	(95% CI)
**Hearing assessment result**										
No	Ref		Ref		Ref		Ref		Ref	
Unilateral	0.2	(−11.7 ~ 12.1)	−4.6	(− 14.0 ~ 4.7)	10.5	(−3.1 ~ 24.2)	−0.6	(−6.7 ~ 5.4)	−5.7	(−15.5 ~ 4.1)
Mild	−4.7	(− 15.5 ~ 6.2)	−15.8	(−23.7 ~ − 8.0)***	3.8	(− 9.0 ~ 16.7)	− 5.4	(− 10.5 ~ − 0.2)*	−7.3	(− 15.7 ~ 1.0)
Moderate or worse	2.1	(− 13.4 ~ 17.5)	−12.7	(− 24.7 ~ − 0.8)*	8.7	(− 9.7 ~ 27.2)	− 7.4	(− 15.4 ~ 0.7)	− 16.0	(− 29.0 ~ − 3.0)*
**Antenatal care visit < 7**										
No	Ref		Ref		Ref		Ref		Ref	
Yes	−2.8	(− 12.6 ~ 6.9)	−4.3	(−11.5 ~ 2.8)	−6.5	(− 17.5 ~ 4.6)	− 5.7	(− 10.4–1.1)*	− 2.5	(− 10.0 ~ 5.0)
**Teenage mother (mother aged < 20)**										
No	Ref		Ref		Ref		Ref		Ref	
Yes	−1.9	(−11.2 ~ 7.4)	2.6	(−4.5 ~ 9.6)	−2.3	(−13.3 ~ 8.6)	−2.3	(−6.6 ~ 2.1)	− 2.1	(− 9.2 ~ 5.0)
**Mother drinking alcohol during pregnancy**										
No	Ref		Ref		Ref		Ref		Ref	
Yes	−0.02	(−14.8 ~ 14.7)	4.3	(−7.0 ~ 15.6)	1.4	(−16.6 ~ 19.3)	0.4	(− 7.2 ~ 8.0)	6.3	(−4.7 ~ 17.2)
**Mother smoking during pregnancy**										
No	Ref		Ref		Ref		Ref		Ref	
Yes	0.5	(−9.4 ~ 10.4)	−5.4	(−13.0 ~ 2.1)	2.6	(−9.1 ~ 14.4)	−3.3	(−8.2 ~ 1.5)	1.4	(−6.4 ~ 9.1)
**Male gender**										
No	Ref		Ref		Ref		Ref		Ref	
Yes	−19.9	(−28.5 ~ − 11.3)***	− 27.8	(−34.0 ~ − 21.7)***	−14.9	(− 24.8 ~ − 4.9)**	−8.6	(−12.6 ~ − 4.7)***	−13.5	(−20.1 ~ − 6.9)***
**Birthweight (gram)**										
≥ 2500	Ref		Ref		Ref		Ref		Ref	
< 2500	2.3	(−11.0 ~ 15.6)	−7.6	(− 16.8 ~ 1.5)	6.4	(−9.2 ~ 22.0)	−3	(−8.6 ~ 2.7)	− 11.7	(−22.3 ~ − 1.1)*
**Preterm birth (< 34 wks GA)**										
No	Ref		Ref		Ref		Ref		Ref	
Yes	1.1	(−10.8 ~ 12.9)	−11.3	(− 19.7 ~ −2.9)	1.2	(− 13.4 ~ 15.8)	−1.5	(−7.2 ~ 4.2)	−5.9	(− 16.3 ~ 4.5)
**Parity**										
1	Ref		Ref		Ref		Ref		Ref	
2–3	8.0	(−1.7 ~ 17.8)	−2.6	(− 10.0 ~ −4.8)	4.2	(−7.5 ~ 15.8)	3.5	(− 1.5 ~ 8.4)	−0.1	(− 7.8 ~ 7.5)
4+	5.1	(−8.2 ~ 18.5)	−7.6	(−17.8 ~ 2.5)	−5.1	(−20.2 ~ 9.9)	− 1.5	(− 7.6 ~ 4.5)	−11.3	(− 22.7 ~ 0.1)
**Apgar 5**										
≥ 7	Ref		Ref		Ref		Ref		Ref	
< 7	−9.7	(−32.9 ~ 13.4)	−19	(−35.2 ~ −2.7)*	−41.6	(−68.3 ~ − 14.9)**	− 3.6	(−13.6 ~ 6.3)	1.6	(−16.1 ~ 19.2)
**Attended preschool**										
No	Ref		Ref		Ref		Ref		Ref	
Yes	2.7	(−9.5 ~ 15.0)	−1.1	(−10.1 ~ 8.0)	10.4	(−3.5 ~ 24.3)	1.5	(−4.7 ~ 7.7)	−0.1	(−9.1 ~ 9.0)
**Average household size^**										
≤ 5	Ref		Ref		Ref		Ref		Ref	
> 5	−2.9	(−38.7 ~ 33.0)	−31.2	(−58.0 ~ −4.3)	− 11.8	(− 51.4 ~ 27.8)	−7.7	(− 23.1 ~ 7.7)	−38.2	(−67.0 ~ − 9.3)*
**Average persons per bedroom^**										
≤ 2	Ref		Ref		Ref		Ref		Ref	
> 2	−5.6	(−45.3 ~ 34.2)	−23.3	(− 55.1 ~ 8.4)	3	(−46.0 ~ 52.1)	1.5	(−17.4 ~ 20.3)	−21.1	(−68.2 ~ 25.9)
**Very remote^**										
No	Ref		Ref		Ref		Ref		Ref	
Yes	−19.0	(− 46.9 ~ 8.9)	−12.5	(−32.4 ~ 7.4)	7.9	(−22.9 ~ 38.8)	−13.8	(−26.4 ~ − 1.1)*	−15.3	(− 35.1 ~ 4.6)
**ESL**										
No	Ref		Ref		Ref		Ref		Ref	
Yes	−3.1	(−23.6 ~ 17.4)	−20.5	(− 36.6 ~ −4.4)*	−34.7	(−57.7 ~ − 11.7)**	−14.9	(−25.2 ~ − 4.7)**	−19.2	(−32.6 ~ − 5.7)*
**Age at Y3 NAPLAN**										
8 yr	Ref		Ref		Ref		Ref		Ref	
< 8	−5.3	(−19.6 ~ 9.0)	−10.8	(− 19.8 ~ − 1.7)*	−13.1	(−28.5 ~ 2.2)	−7.3	(− 12.2 ~ − 2.3)**	−20.3	(− 31.2 ~ − 9.3)***
≥ 9	− 10.7	(− 26.7 ~ 5.3)	11.8	(−1.2 ~ 24.7)	22.7	(2.4 ~ 43.0)*	3.9	(− 5.2 ~ 13.0)	0.9	(−11.7 ~ 13.5)
**Year 2 attendance**										
≥ 80%	Ref		Ref		Ref		Ref		Ref	
60–79%	−14.8	(−25.3 ~ −4.3)*	− 28.7	(−36.9 ~ − 20.5)***	−21.7	(− 34.1 ~ −9.3)**	−11.7	(− 17.1 ~ −6.2)***	−16.4	(− 24.1 ~ −8.7)***
< 60%	−26.8	(− 38.9 ~ − 14.7)***	− 52.7	(− 61.4 ~ − 43.9)***	− 35.9	(− 49.5 ~ − 22.2)***	− 23.1	(− 28.7 ~ − 17.5)***	−29.6	(− 38.5 ~ − 20.7)***

^ Community level variables

* *p* < 0.05; ** *p* < 0.005; *** *p* < 0.0005

Note: NAPLAN: National Assessment Program – Literacy and Numeracy; Unadj COEF: unadjusted coefficient; ESL: Speaking English as second language

As the estimated association in univariate regression was strongest for Writing, the multivariate regression model was first developed for this domain, and then used as a base model for other domains. The final model for Writing was subsequently demonstrated to be the best fit for all domains. In addition to the variables retained to control for confounding (sex, year of the NAPLAN test, age at Year 3 NAPLAN test, Year 2 attendance, attended preschool, and two community level variables: living in a very remote community and average household size > 5 persons), other covariates included in the final, parsimonious models were birthweight< 2500 g (LBW) and speaking English as second language (ESL). The associations found in the univariate analyses remained in the multivariate fixed effects models, after controlling for a range of potential confounding variables and covariates (Table 2). Compared with children with normal hearing, those with mild HI on average scored lower in Writing and Spelling by an adjusted COEFs of − 15.0 points (95% CI: − 22.4 to − 7.6, *p* < 0.0005) and − 5.0 points (95% CI: − 9.6 to − 0.3, *p* = 0.037), respectively; and those with moderate or worse HI scored lower in Writing and Numeracy by − 12.8 points (95% CI: − 24.2 to − 1.4, *p* = 0.028) and − 15.0 points (95% CI: − 27.4 to − 2.6, *p* = 0.018), respectively. Being “male’ was associated with poorer academic achievement in all five domains with the adjusted COEFs ranging from − 8.7 points (in Spelling) to − 27.9 points (in Writing). Year 2 attendance rates was also a strong predictor for poorer outcomes, and the effect sizes for the attendance < 60% category was substantially greater than that for the 60–79% category. Other variables showing evidence for an association in some but not all domains included LBW (in Numeracy), average household size> 5 (in Writing and Numeracy), living in very remote communities (in Spelling), ESL (in all domains except Reading) and age at Year 3 NAPLAN< 8 years (in Writing, Spelling and Numeracy). Children that were 9 years or older were found to have higher scores in Grammar compared to the reference category (adjusted COEF: 22.2, 95% CI: 1.9–42.6, adjusted *p* = 0.032).

**Table 2:** Results of multivariate regression analyses for the association between the predictor and control variables, and the scale scores of NAPLAN Year 3 tests in five domains, Northern Territory Aboriginal children, NAPLAN tests from 2009 to 2016

Variable	Reading		Writing		Grammar		Spelling		Numeracy	
	Adj COEF	(95% CI)	Adj COEF	(95% CI)	Adj COEF	(95% CI)	Adj COEF	(95% CI)	Adj COEF	(95% CI)
**Hearing assessment result**										
No	Ref		Ref		Ref		Ref		Ref	
Unilateral	−0.5	(−12.0 ~ 11.0)	−3.4	(− 12.3 ~ 5.5)	8.3	(−5.0 ~ 21.6)	0.1	(−5.5 ~ 5.8)	−3.0	(−12.6 ~ 6.5)
Mild	−5.7	(−16.4 ~ 5.1)	−15.0	(−22.4 ~ −7.6)***	1.1	(− 11.5 ~ 13.7)	− 5.0	(−9.6 ~ − 0.3)*	−5.2	(−13.5 ~ 3.0)
Moderate or worse	0.5	(−14.6 ~ 15.6)	−12.8	(− 24.2 ~ − 1.4)*	5.1	(− 12.5 ~ 22.6)	−6.6	(− 14.1 ~ 1.0)	−15.0	(− 27.4 ~ − 2.6)*
**Male gender**										
No	Ref		Ref		Ref		Ref		Ref	
Yes	−18.2	(−26.7 ~ −9.7)***	− 27.9	(−34.1 ~ − 21.8)***	−14.9	(−24.8 ~ −5.0)**	−8.7	(− 12.6 ~ −4.8)***	−13.5	(−20.1 ~ −6.8)***
**Birthweight (gram)**										
≥ 2500	Ref		Ref		Ref		Ref		Ref	
< 2500	2.4	(−10.6 ~ 15.3)	−7.6	(− 16.8 ~ 1.5)	6.4	(−9.2 ~ 21.9)	−3.0	(−8.6 ~ 2.6)	− 11.7	(−22.3 ~ −1.1)*
**Attended preschool**										
No	Ref		Ref		Ref		Ref		Ref	
Yes	−6.8	(−19.6 ~ 5.9)	3.5	(−5.5 ~ 12.5)	−7.1	(−21.5 ~ 7.2)	−2.9	(−9.0 ~ 3.1)	2.5	(−7.2 ~ 12.3)
**Average household size^**										
≤ 5	Ref		Ref		Ref		Ref		Ref	
> 5	−4.7	(−36.8 ~ 27.5)	−31.2	(−58.1 ~ −4.3)*	−11.9	(− 51.7 ~ 27.8)	−7.8	(−23.1 ~ 7.6)	− 38.2	(−67.0 ~ −9.3)*
**Very remote^**										
No	Ref		Ref		Ref		Ref		Ref	
Yes	−12.4	(−38.9 ~ 14.1)	−12.4	(−32.4 ~ 7.6)	8.3	(−22.7 ~ 39.2)	−13.5	(− 26.2 ~ −0.9)*	−15.3	(− 35.2 ~ 4.5)
**ESL**										
No	Ref		Ref		Ref		Ref		Ref	
Yes	−5.2	(−24.2 ~ 13.8)	−20.6	(−36.7 ~ −4.5)*	−34.6	(− 57.6 ~ −11.6)**	−14.9	(−25.1 ~ − 4.7)**	−19.2	(− 32.7 ~ − 5.7)*
**Age at Y3 NAPLAN**										
8 yr	Ref		Ref		Ref		Ref		Ref	
< 8	−3.8	(−17.6 ~ 10.1)	−10.7	(−19.8 ~ −1.7)*	−13.1	(−28.4 ~ 2.2)	−7.2	(−12.1 ~ − 2.3)**	−20.3	(−31.2 ~ −9.4)***
≥ 9	−4.8	(−20.5 ~ 11.0)	11.8	(−1.1 ~ 24.8)	22.2	(1.9 ~ 42.6)*	3.6	(−5.2 ~ 12.7)	1.1	(−11.5 ~ 13.8)
**Year 2 attendance**										
≥ 80%	Ref		Ref		Ref		Ref		Ref	
60–79%	−12.5	(−22.8 ~ −2.2)*	−28.7	(−36.8 ~ − 20.5)***	−21.7	(− 34.1 ~ −9.3)**	−11.6	(−17.1 ~ −6.1)***	−16.4	(−24.1 ~ −8.8)***
< 60%	−26.1	(− 38.0 ~ −14.2)***	−52.7	(− 61.4 ~ −43.9)***	−35.8	(− 49.5 ~ − 22.1)***	−23.1	(− 28.7 ~ − 17.5)***	−29.6	(−38.6 ~ − 20.7)***
**Model statistics**										
Number of children	1992		2045		2066		2066		1953	
Adjusted R-squared	0.335		0.326		0.264		0.290		0.245	

^ Community level variables

* *p* < 0.05; ** *p* < 0.005; *** *p* < 0.0005

Note: NAPLAN: National Assessment Program – Literacy and Numeracy; Adj COEF: adjusted coefficient; ESL: Speaking English as second language

There was no evidence of associations between unilateral hearing loss and academic outcomes.

## Discussion

This study provides robust evidence for an independent association between HI and early years academic achievement for Aboriginal children living in remote NT communities. To our knowledge, this is the first Australian study using population-level, audiometrically assessed hearing data for Aboriginal children to investigate the impact of HI on educational outcomes. Our findings also provide further evidence for health determinants of educational attainment [41].

Our analyses showed that, compared with children from the same population with normal hearing, those with bilateral HI had lower scores in three of the five NAPLAN domains: Writing, Spelling and Numeracy. For Writing, the score difference between ‘normal hearing’ and ‘mild HI’ categories as estimated in the multivariate model, 15.0 points, represented 7.3% of the mean score of the study cohort; and for the difference between ‘normal hearing’ and ‘moderate or worse HI’, it was 6.3%. For Spelling, the score difference for ‘mild HI’ was 2.1% of the study cohort’s mean score; and for Numeracy, it was ‘moderate or worse HI’ that showed an association with poorer NAPLAN outcomes, and the difference was equivalent to 6.3% of the study cohort’s mean score, compared with children assessed as having normal hearing. These differences in scale scores were estimated while holding the covariates selected from the multivariate model constant, and thus demonstrated not only evidence for an association but also the magnitude of the independent impact of HI on academic achievement for children aged around 8 years old.

Notably, the effect size of HI was modest compared with other variables included in the final regression model. For example, for Writing, the adjusted coefficient for ‘mild HI’ or ‘moderate or worse HI’ was − 15.0 and − 12.8 points respectively but was − 27.9 points for boys (compared with girls) and − 20.6 for ESL children (compared with ‘non-ESL’ children). The adjusted coefficients for Year 2 attendance rates were even greater: − 28.7 points for ‘60–79%’ and a substantially higher − 52.7 points for the lower attendance of ‘< 60%’. Compared with the mean score for the study cohort of 204.8 points, these were equivalent to 14.0 and 25.7% of the score. The community level factor representing housing crowdedness, ‘average household size >5 persons’ also showed a negative correlation with the outcome measure in Writing and Numeracy with effect sizes two times or greater than those produced by HI. These findings are consistent with previous studies conducted in the NT and reinforce the past findings that there are multiple and overlapping influences on academic outcomes for Aboriginal children [24, 42]. Finally, the modest effect sizes of HI may be the reason for the lack of evidence for an increase of effect with increasing degree of HI, as judged by the overlapping 95% confidence intervals for estimates. It may also be that at this young age any degree of HI may have a similar impact on the outcome. Further research is needed to detect the differential impacts of varying levels of HI.

These findings of the importance of HI have a number of implications for both health and education sectors. Public health programs for primary prevention (such as pneumococcal vaccination programs to reduce OM prevalence), secondary prevention (such as early detection and treatment of OM and HI through regular clinical and audiometric examination) and tertiary treatment (surgical interventions for OM) implemented to reduce the incidence and prevalence of OM and the associated HI can also be expected to reduce the risk of poorer academic achievement in primary school, both directly and indirectly, by reducing HI’s impact on other factors that also impact on academic achievement.

From an educational point of view, the impact of fluctuating, mostly mild to moderate conductive HI on children’s learning and academic performance has long been seen as questionable, based on inconclusive research findings among non-Indigenous populations. However, a greater proportion of Aboriginal children experience more severe middle ear infection and the associated HI earlier and for longer periods than other Australian children. They also more often live in crowded, noisy housing conditions and experience other forms of disadvantage that can compound the impact of HI. This means that it is crucial to inform health and educational policy and practice that research examining the impacts of HI needs to be undertaken with Aboriginal populations. Our findings support the implementation of early and active detection of students with HI, which can facilitate timely and appropriate educational support to those affected. Educational support for such children may include installing suitable sound amplification hardware in classrooms, improving classroom acoustics and training teachers to improve their expertise in supporting hearing impaired students [12, 43]. Other research points to the need for teachers to be aware of the adverse psychosocial experiences and higher risk of school behavioural problems related to Aboriginal children’s HI [44, 45]. Communication between health and educational professionals is important so that teachers are aware of those children who may need additional support at school. Teachers can also assist parents to understand likely communication issues at home that may contribute to parent-child conflict. As well as teachers having a crucial role in improving school learning they may also be able to support OM treatment adherence and, where indicated, the possibility of surgical or medical treatment to improve HI.

The major strength of our study is the use of audiometrically determined HI, and not clinical diagnosis of OM, as the main predictor variable. This eliminates the uncertainty and potential misclassification that can arise for those children with OM and normal hearing. Another strength is the use of data from a range of health and education administrative datasets, which allowed for the inclusion of potential confounders to more accurately estimate the independent association between the predictor and the targeted outcome variables. Furthermore, our use of NAPLAN scale scores as the outcome measure avoided the loss of precision arising when dichotomising continuous data [46]. This is particularly relevant when the NMS for each domain, based on a national population, is applied to a population in which a high proportion of Aboriginal students scored below the NMS (for example, 46.6% of Year 3 Aboriginal students scored below the NMS in Numeracy in 2016 [30]).

Our study has a number of limitations. First, children with records in the RHA dataset were not representative of all NT Aboriginal children. Although the ear health service provided by the NT Outreach Hearing Health Program was free and delivered to remote communities, access was by referral through the local clinic and was not universal [20]. There was evidence of differences between the study cohort and children for whom there was no hearing data. The differences included geographic distribution, partly because of the absence of urban Aboriginal children (from the Darwin area) in the study cohort, which overlapped with other differences including higher NAPLAN scores, across all five domains, for the comparison group compared with the study cohort. Second, the low adjusted R squared estimates, in the final regression models, indicate that the models explain less than a third of the variance in the academic results and that HI had only a modest contribution to these outcomes. Further research with additional explanatory factors is needed to develop a comprehensive explanation of academic achievement for this population. Third, school attendance data was only available for government schools, however the impact will be minor as there are only a small number of non-government schools in very remote NT communities [47]. Measures have now been taken to facilitate the inclusion of all NT school data for future studies. Fourth, due to constraints on the availability and timing of the hearing assessment it was necessary to use each child’s first audiometry result for analysis, with the assumption that the result was indicative of the child’s long-term hearing status. Given that the severity of HI may change with time, it is likely that our approach had introduced some misclassification. Some children classified with HI might have their hearing return to normal after treatment, while others assessed to have normal hearing may have developed HI after the assessment. Taking into consideration the persisting and recurrent nature of OM in this population, we believe the overall impact of such misclassifications would have led to underestimation of the true effect size of HI on the outcome. A final limitation may be the validity of NAPLAN tests. NAPLAN tests were developed with the advice of specialist officers in Indigenous education, English as a second language and special needs education, [48] and have been widely used as measures of academic performance for both Aboriginal and non-Aboriginal students (for example, [24, 31] [49];). However there remains concern for the validity of NAPLAN for assessing academic performance for Aboriginal students. For example, Wigglesworth et al. [50] commented that a lack of Western cultural exposure and knowledge can affect Aboriginal students’ performance in some literacy test items and the worded numeracy questions. Our study has avoided this potential bias by reporting differences in results between groups of Aboriginal children of similar background and not with non-Aboriginal children.

## Conclusions

The evidence produced by this study indicates that Aboriginal children with a history of hearing impairment are at higher risk of poorer academic achievement in Year 3 of primary school. To improve the overall academic achievement in Aboriginal children, interventions need to incorporate the prevention and treatment of otitis media and strategies to support affected children. Our study also demonstrates the utility of cross-agency data linkage that combines hearing assessment data with other administrative datasets in facilitating comprehensive investigations not possible using stand-alone datasets or survey methods.

## Supplementary information

**Additional file 1.**

## Data Availability

The study datasets contain sensitive personal information and are held on a secure cloud-based server with restricted access. Access requires the approval of the ethics committee and data custodians.

## Abbreviations

NT: Northern Territory
HI: Hearing impairment
OM: Otitis media
OME: Otitis media with effusion
NAPLAN: National Assessment Program – Literacy and Numeracy
SA: South Australia
RHA: Remote Hearing Assessment
dB: decibel
HL: Hearing loss
ESL: English as second language
IRSD: Index of Relative Socio-economic Disadvantage
SLA: Statistical local area
ARIA+: Accessibility and Remoteness Index of Australia
