# Supplementary material for: The impact of hearing impairment on early academic achievement in Aboriginal children living in remote Australia: a data linkage study
Source: BMC Public Health. 2020 Oct 7;20:1521. doi: 10.1186/s12889-020-09620-6 (PMC7542869; doi:10.1186/s12889-020-09620-6)
Supplement: Supplementary file 1 — Additional file 1. [file 12889_2020_9620_MOESM1_ESM.docx]

Appendix Table 1: Admission diagnoses and surgical procedures related to otitis media and their International Classification, 10th Revision- Australian Modification codes used in the exclusion criteria

| ICD-10AM Code | Diagnosis / Procedure |
| --- | --- |
| Diagnosis |  |
| H65 | Nonsuppurative otitis media |
| H66 | Suppurative and unspecified otitis media |
| H72 | Perforation of tympanic membrane |
| Procedure |  |
| 41527-00 | Myringoplasty, transcanal approach |
| 41530-00 | Myringoplasty postaural or endaural approach |
| 41533-01 | Myringoplasty with atticotomy |
| 41542-00 | Myringoplasty with ossicular chain reconstruction |
| 41551-00 | Mastoidectomy by intact canal wall technique with myringoplasty |
| 41554-00 | Mastoidectomy by intact canal wall technique with myringoplasty and ossicular chain reconstruction |
| 41560-00 | Modified radical mastoidectomy with myringoplasty |
| 41560-01 | Radical mastoidectomy with myringoplasty |
| 41563-00 | Modified radical mastoidectomy with myringoplasty and ossicular chain reconstruction |
| 41563-01 | Radical mastoidectomy with myringoplasty and ossicular chain reconstruction |
| 41626-00 | Myringotomy, unilateral |
| 41626-01 | Myringotomy, bilateral |
| 41632-00 | Myringotomy with insertion of tube, unilateral |
| 41632-01 | Myringotomy with insertion of tube, bilateral |
| 41635-01 | Excision of lesion of middle ear with myringoplasty |
| 41638-01 | Excision of lesion of middle ear with myringoplasty and ossicular chain reconstruction |
| 41789-00 | Tonsillectomy without adenoidectomy |
| 41789-01 | Tonsillectomy with adenoidectomy |
| 41801-00 | Adenoidectomy without tonsillectomy |
| 90114-00 | Other procedures on eardrum or middle ear |

Appendix Table 2: Comparison between the study cohort and comparison group on selected control and outcome variables

| **Variables** | **% of all** | **Study Cohort (n=2208)** | | **Comparison Group (n=3383)** | | **p value** |
| --- | --- | --- | --- | --- | --- | --- |
|  |  | **%** | **(95% CI)** | **%** | **(95% CI)** |  |
| **Control variables** |  |  |  |  |  |  |
| Sex (being male) | 50.3 | 50.4 | (48.3-52.4) | 50.2 | (48.5-51.9) | 0.918 |
| Age ≥ 8 years | 89.0 | 88.2 | (86.8-89.5) | 89.5 | (88.5-90.6) | 0.113 |
| Very remote | 76.1 | 92.4 | 91.3-93.5 | 65.4 | (63.8-67.0) | <0.001 |
| Average household size>5 | 25.4 | 28.6 | (26.7-30.5) | 23.3 | (21.9-24.7) | <0.001 |
| Average persons per bedroom>2 | 10.2 | 11.5 | (10.2-12.9) | 9.3 | (8.3-10.3) | 0.007 |
| Antenatal care visits < 7 | 34.5 | 35.5 | (33.5-37.5) | 33.8 | (32.2-35.4) | 0.196 |
| Low birthweight | 11.9 | 12.5 | (11.1-13.9) | 11.4 | (10.4-12.5) | 0.232 |
| Speak English as 2nd language | 79.7 | 91.8 | (90.7-92.9) | 71.8 | (70.3-73.3) | <0.001 |
| Attended preschool | 76.9 | 78.8 | (77.1-80.5) | 75.6 | (74.2-77.1) | 0.006 |
| Year 2 attendance rate < 80% | 63.7 | 68.5 | (66.6-70.5) | 60.5 | (58.8-62.1) | <0.001 |
|  |  |  |  |  |  |  |
| **Outcome variables** |  |  |  |  |  |  |
| **Scale score (mean)** | **Mean of all** | **Mean** | **(95% CI)** | **Mean** | **(95% CI)** | **p value** |
| Reading | 219.6 | 197.2 | (192.2-202.1) | 234.3 | (230.2-238.4) | <0.001 |
| Writing | 225.3 | 204.8 | (201.2-208.3) | 238.6 | (235.1-242.1) | <0.001 |
| Grammar | 196.3 | 173.6 | (168.1-179.1) | 211.1 | (206.5-215.7) | <0.001 |
| Spelling | 250.9 | 235.6 | (233.4-237.9) | 260.8 | (258.3-263.4) | <0.001 |
| Numeracy | 252.5 | 238.8 | (235.2-242.4) | 261.3 | (258.3-264.3) | <0.001 |
